# Prenatal diagnosis and preimplantation genetics testing of 3M syndrome in a Chinese family with novel biallelic variants of *CUL7*

**DOI:** 10.1002/mgg3.2284

**Published:** 2023-10-25

**Authors:** Xueqian Wang, Yaqiong He, Xiaorong Wang, Xiangtian Kong, Yunying Lin, Yejie Yao, Yi Huang

**Affiliations:** ^1^ Department of Prenatal Screening and Diagnosis Center Affiliated Maternity and Child Health Care Hospital of Nantong University Nantong Jiangsu China; ^2^ Nantong Institute of Genetics and Reproductive Medicine Affiliated Maternity and Child Health Care Hospital of Nantong University Nantong Jiangsu China; ^3^ Center for Reproductive Medicine, Ren Ji Hospital, School of Medicine Shanghai Jiao Tong University Shanghai China; ^4^ Shanghai Key Laboratory for Assisted Reproduction and Reproductive Genetics Shanghai China; ^5^ Center for Reproductive Medicine Affiliated Maternity and Child Health Care Hospital of Nantong University Nantong Jiangsu China

**Keywords:** 3M syndrome, noncanonical splice site, preimplantation genetics test, prenatal diagnosis, RT‐PCR

## Abstract

**Background:**

3M syndrome is a rare autosomal recessive developmental disorder characterized by pre and postnatal growth deficiency, dysmorphic facial features, and normal intelligence. 3M syndrome should be suspected in a proband with a combination of characteristic or recognizable dysmorphic features. The diagnosis of 3M syndrome could be confirmed by identifying biallelic variants in *CUL7*, *OBSL1*, or *CCDC8*.

**Methods:**

Whole‐exome sequencing (WES) was performed to identify genetic causes. Reverse‐transcription polymerase chain reaction (RT‐PCR) was performed to detect aberrant splicing events. Haplotypes were constructed using multiplex PCR and sequencing. Variants of the parental haplotype and target likely pathogenic variants were detected by PCR and Sanger sequencing from the embryos. Copy number variant (CNV) detection was performed by next‐generation sequencing.

**Results:**

We present the case of a nonconsanguineous Chinese couple with one abnormal pregnancy, where the fetus showed 3M phenotypes of shortened long bones. WES identified two novel heterozygous mutations in *CUL7*: NM_014780.5:c.354del (p.Gln119ArgfsTer52) and NM_014780.5:c.1373‐15G>A. RT‐PCR from RNA of the mother's peripheral blood leucocytes showed that c.1373‐15G>A caused the insertion of a 13‐bp extra intron sequence and encoded the mutant p.Leu459ProfsTer25. Both variants were classified as likely pathogenic according to ACMG/AMP guidelines and Clinical Genome Resource specifications. During genetic counseling, the options of prenatal diagnosis through chorionic villus sampling or amniocentesis, adoption, sperm donation, and electing not to reproduce, as well as preimplantation genetic testing for monogenic disorders (PGT‐M), were discussed. The couple hopes to conceive a child of their own and refused to accept the 25% risk during the next pregnancy and opted for PGT‐M. They finally successfully delivered a healthy baby through PGT‐M.

**Conclusion:**

This study expanded the mutation spectrum of *CUL7*, detected the aberrant splicing event of *CUL7* via RT‐PCR, constructed the haplotype for PGT‐M, and demonstrated the successful delivery of a healthy baby using PGT‐M.

## Graphical abstract

In this study, we discovered a biallelic variant of CUL7 in a fetus with shortened long bones. Reverse transcriptase PCR analysis of lymphocytes from the carrier confirmed the variant resulted in aberrant splicing, leading to the reclassification of the intronic variant c.1373‐15G) A as likely pathogenic. After genetic counseling was provided to the couple, the couple chose preimplantation genetic testing for monogenic disorders for their next pregnancy, which resulted in the successful birth of a healthy baby. Our study highlights the application of molecular methods in a clinical setting.

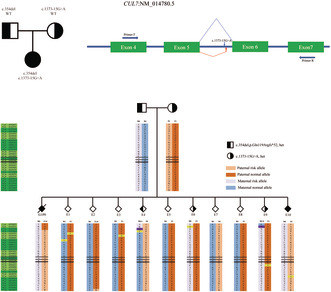

## 1 INTRODUCTION

3M syndrome is an autosomal recessive disorder characterized by pre‐ and postnatal growth deficiency, dysmorphic facial features, and normal intelligence. Affected individuals may exhibit features, including a short broad neck, dolichocephaly, triangular face, midface retrusion, prominent trapezius, malformed sternum, slender long bones, short thorax, tall vertebral bodies, small pelvic bones, prominent heels, joint hypermobility, and hypogonadism in males. 3M syndrome was named after the three investigators who first described it (Miller et al., 1975). The prevalence of 3M syndrome has not yet been clearly defined, but pathogenic variants of three genes, namely, *CUL7* (3M1, MIM #273750), *OBSL1* (3M2, MIM #612921), and *CCDC8* (3M3, MIM #614205), were responsible for 77.5%, 16%, and <5% of 3M syndrome cases, respectively (Hanson et al., 2009, 2011; Huber et al., 2009, 2011).

*CUL7*, located on 6p21.1, encodes a component of an E3 ubiquitin‐protein ligase complex, that plays a crucial role in microtubule dynamics and genome integrity (Yan et al., 2014). Cul7^tm1a(EUCOMM)Wtsi^/Cul7^+^ mice exhibit abnormal bone mineralization, decreased body weight, and reduced bone mineral density. Additionally, Cul7^tm1Jdec^/Cul7^tm1Jdec^ mice exhibit severe fetal growth restriction and perinatal death. Histology revealed vascular defects in both the embryo and the placenta (Arai et al., 2003). Moreover, Cul7^tm1Zqp^/Cul7^tm1Zqp^ mice exhibits fetal growth retardation and lethality (Xu et al., 2008). However, the 3M syndrome in human mainly affected bones and could survived postnatally which is different from the phenotype of homozygosity knock out in mice. As a result, the mechanism by which *CUL7* deficiency causes 3M syndrome remains unclear. The Clinical Genome (ClinGen) Resource has not yet published gene‐disease clinical validity curation results for *CUL7*. The Gene Curation Coalition website has received four submissions, with two rated as “Definitive,” one as “Strong,” and one as “Supportive.” Multiple studies have identified >50 loss of function (LOF) variants in *CUL7* in the HGMD database (professional 2022.4).

Here, we describe the case of a Chinese family whose first fetus was prenatally diagnosed with 3M syndrome due to two novel *CUL7* variants. Reverse‐transcription polymerase chain reaction (RT‐PCR) was performed to confirm the aberrant splicing event caused by the noncanonical splice site variant. Preimplantation genetic testing for monogenic disorders (PGT‐M) was conducted to aid the delivery of a healthy baby. Our study expands the spectrum of *CUL7* mutations and, for the first time, demonstrates the effectiveness of PGT‐M in preventing 3M syndrome.

## 2 MATERIALS AND METHODS

### 2.1 Ethical compliance

Studies involving human participants were reviewed and approved by the Medical Ethics Committee of the Affiliated Maternity and Child Health Care Hospital of Nantong University (Y2020071). Amniocytes and peripheral blood leukocytes were collected after obtaining a written informed consent.

### 2.2 Whole‐exome sequencing (WES)

Genomic DNA was extracted from fetal amniocytes and peripheral blood leukocytes of the parents using the salt fractionation method (Magen, Guangdong, China). Exome capture was performed using xGen Exome Research panel v1 (Integrated DNA Technologies, Coralville, IA, USA), and sequencing was conducted on NovaSeq 6000 (Illumina, San Diego, CA). The sequences were aligned to a human reference sequence (NCBI Genome build GRCh37) using the Burrows–Wheeler Aligner. The Genome Analysis Toolkit pipeline was used to detect single‐nucleotide (SNPs) and indel polymorphisms. Prioritization was given to variants that were previously reported, considered LOF (nonsense, frameshift, or splice site mutations), or absent/rare in gnomAD. The candidate gene list was then narrowed down based on abnormal long bone morphology (Human Phenotype Ontology [HP]: 0011314). Variant interpretation was performed in accordance with the ACMG/AMP guidelines and ClinGen specifications (Richards et al., 2015; Zhang et al., 2020).

### 2.3 RT‐PCR

Peripheral blood leukocytes were isolated from the mother using a lymphocyte separation medium (TBDscience, Tianjin, China). RNA was extracted using the RNA‐easy isolation reagent (Vazyme Biotech, Nanjing, China). Reverse transcription was performed using Goldenstar™ RT6 cDNA Synthesis Kit (Tsingke Biotech, Beijing, China). The fragment between exons 4 and 7 was amplified using the following primers: forward 5′‐GTTCGCAAGTGGCAATACCT‐3′, reverse 5′‐CAAGTCCTGGGCCAATTCTA‐3′. The fragment was then cloned into a vector using the 5 min. TA/Blunt‐Zero Cloning Kit (Vazyme Biotech, Nanjing, China) and sequenced with the M13 forward primer.

### 2.4 Haplotype construction

Preliminary experiments on genomic DNA were conducted before PGT. Pathogenic variants and linked SNPs of target genes were identified, and haplotypes were constructed using parental blood and DNA from the proband. SNPs within the 2‐Mb range on each side of the target gene, with a minor allele frequency of >0.2 in the 1000 Genomes Project, were selected. Generally, the number of SNPs was >200. Forward and reverse primers were designed using Primer 5.0 software and synthesized, with amplicon sizes ranging between 125 and 275 bp (Table S1). SNP haplotypes were constructed in collaboration with Peking Jabrehoo Med Tech. First, multiplex PCR was performed to capture the coding region of the target gene and relevant SNPs. Different label sequences (barcodes) were added to multiple samples, followed by library preparation using the Illumina standard process. Sequencing was performed using the MiSeq Sequencing System (Illumina) with an average depth of >100X. The sequencing data were analyzed using the software developed by Peking Jabrehoo Med Tech with the human reference genome GRCh37/hg19.

### 2.5 Assisted reproductive technology procedure and embryo trophectoderm biopsy

Controlled ovarian stimulation was performed using a gonadotropin‐releasing hormone (GnRH) antagonist. Intracytoplasmic sperm injection (ICSI), blastocyst culture, trophectoderm (TE) biopsy, and blastocyst transfer were conducted at the Reproductive Medicine Center of Renji Hospital affiliated with Shanghai Jiao Tong University School of Medicine, according to the standard protocol (Alfarawati et al., 2011; McArthur et al., 2005). In this cycle, 18 oocytes were obtained, and 10 embryos finally developed into blastocysts. TE cells were biopsied on day 5 or 6 after insemination. A total of 5–8 biopsied cells from the TE were transferred into 4.5‐μL lysis buffer in 0.2‐μL PCR tubes for whole‐genome amplification.

### 2.6 Genetic testing

Blastocyst trophectoderm cells were amplified using the REPLI‐g Single Cell Kit (QIAGEN, Hilden, Germany) according to the manufacturer's instructions. PGT‐M was conducted to test for mutated sites and linked SNPs, and Peking Jabrehoo Med Tech was used for haplotype construction. The pathogenic variants/SNPs of the target genes were amplified, followed by Sanger sequencing (Figure S1). The genotypes of the embryos were determined based on the results of pathogenic variant and haplotype analysis (Harton et al., 2011).

PGT for aneuploidy (PGT‐A) was performed with NGS‐based CNV sequencing on the Illumina MiSeqDx platform, which indicated >4 Mb CNV and 30%–70% mosaicism.

### 2.7 Blastocyst transfer and clinical outcome

One euploid embryo free of parental pathogenic variants of *CUL7* was transferred. To verify the results of PGT‐M, amniocentesis was performed at 18 weeks of gestation.

## 3 RESULTS

### 3.1 Clinical phenotype

**FIGURE 1:**
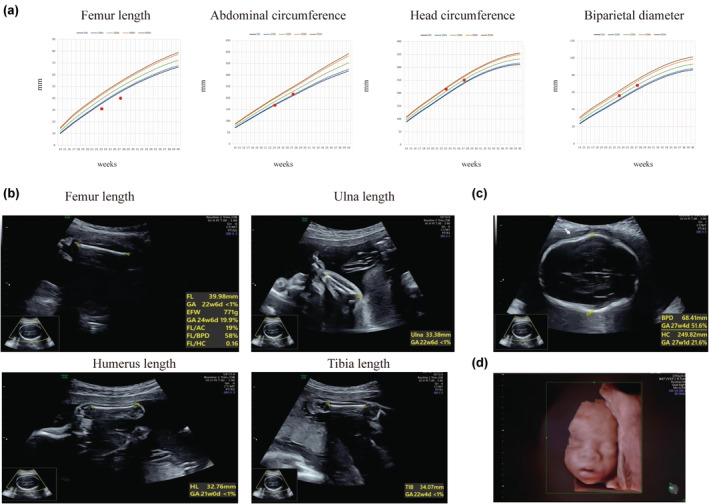
Growth parameters of the affected fetus. (a) The femur length, abdominal circumference, head circumference, and biparietal diameter of the fetus at 23 + 3 and 27 + 2 weeks. (b) Ultrasound image of the fetus at 27 + 2 weeks showing decreased humerus, ulnar, and tibial length. (c) Unilateral temporal bone depression arrow. (d) Facial features of the fetus.

A 25‐year‐old woman, gravida 1 para 0, received regular prenatal care until 23 + 3 weeks of gestation when a prenatal ultrasound revealed that her fetus had a short femur measuring 31 mm (−3.76 SD), an abdominal circumference of 168 mm (−1.76 SD), a head circumference of 215 mm (0.908 SD), a biparietal diameter of 56 mm (0.018 SD), and an estimated fetal weight of 409 g (−2.6. SD). Additionally, short nasal bones (<5%) were observed. At 27 + 2 weeks of gestation, the femur length was 40 mm (−4 SD), the abdominal circumference was 216 mm (−1.13 SD), the head circumference was 250 mm (−0.29 SD), the biparietal diameter was 68 mm (−0.156 SD), and the estimated fetal weight was 776 g (−2.45 SD; Figure 1a). Other bones were also affected, with the humerus length measuring 33 mm (−5.81 SD), the ulnar length measuring 33 mm (−4.8 SD), the tibial length measuring 34 mm (−4.81 SD), and the foot length measuring 42 mm (−3.52 SD; Figure 1b; reference population of Asian and Pacific Islanders from the National Institute of Child Health and Human Development Fetal Growth Study). Unilateral temporal bone depression was also observed.(Figure 1c) Facial features were carefully examined, and no obvious abnormalities were observed. (Figure 1d) Amniocyte karyotyping, chromosomal microarray (Affymetrix 750 k), and FGFR3 c.1138 sequencing were performed, but no aneuploidy, >100‐kb deletion, or >500‐kb duplication CNV or *FGFR3* mutations were detected. The parents decided to terminate the pregnancy at 27 + 5 weeks of gestation, and a female fetus was delivered.

### 3.2 Molecular findings

**FIGURE 2:**
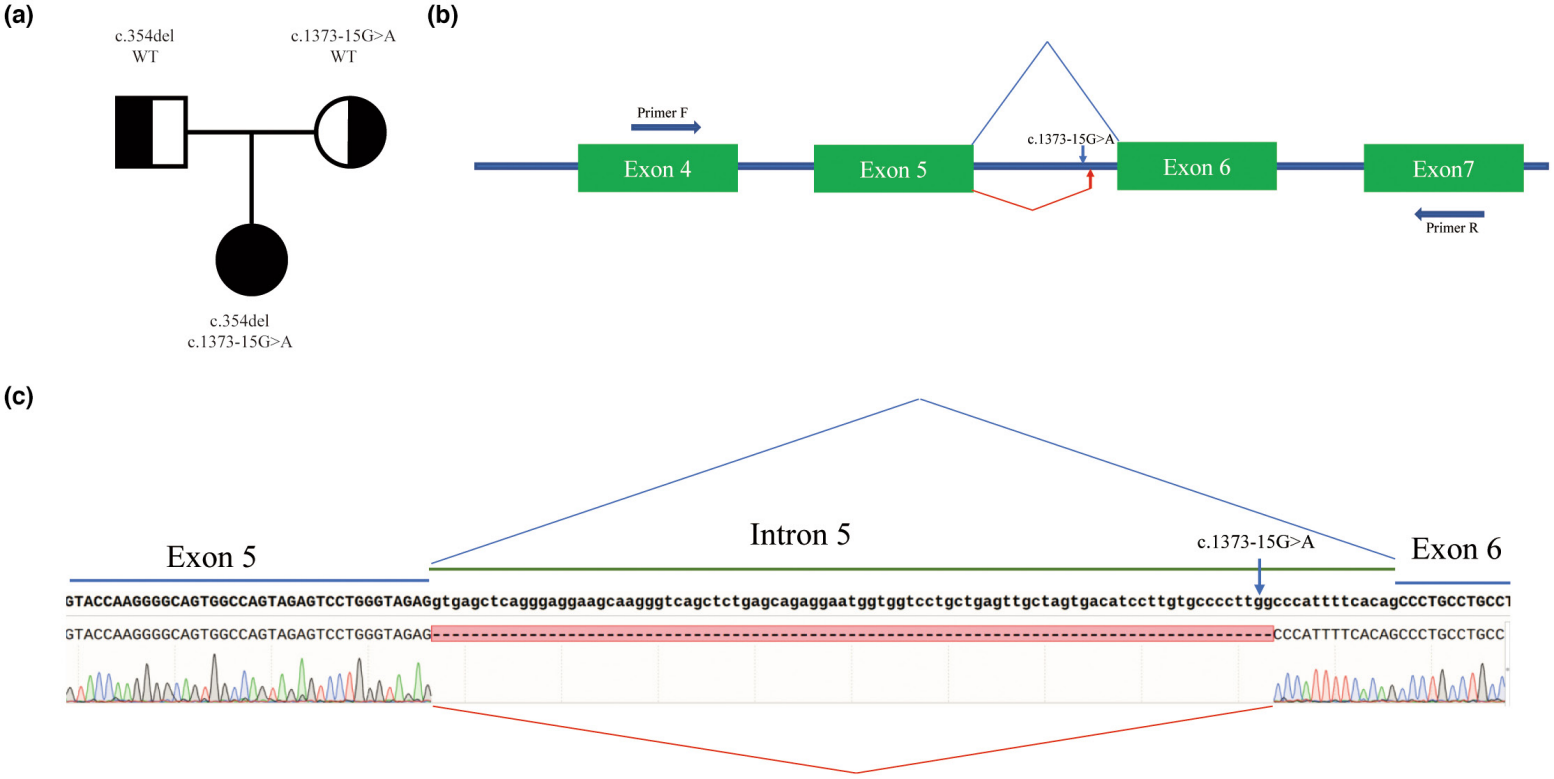
Molecular findings of the family members. (a) Pedigree of the family. (b) Schematic representation of the aberrant splice event. (c) Sanger sequencing results of the reverse‐transcription polymerase chain reaction product.

Using trio‐WES, two compound heterozygous candidate variants were identified in CUL7. NM_014780.5:c.354del (p.Gln119ArgfsTer52) in exon 2 was inherited from the father, and NM_014780.5:c.1373‐15G>A in intron 5 was inherited from the mother (Figure 2a). The c.354del variant is absent in gnomAD and has not been reported in the literature. The deletion of one base pair was predicted to cause a frameshift and nonsense‐mediated decay. Therefore, c.354del was classified as likely pathogenic based on PVS1 and PM2_surpporting. c.1373‐15G>A is a noncanonical splicing variant absent in gnomAD. Splicing AI predicted an acceptor gain at position −2 bp with a score of 0.96, while the formation of a de novo splice site at position c.1373‐13 was predicted as “class 5” by the Varseak website (https://varseak.bio/). Aberrant splicing events were confirmed using RT‐PCR and Sanger sequencing. A frameshift and premature stop were introduced by the insertion of 13‐bp extra intron sequence from the normal transcript NM_014780.5: c.1375_1376insCATTTTCACAGCC (p.Leu459ProfsTer25) (Figure 2b,c). Therefore, c.1373‐15G>A was classified as likely pathogenic based on PVS1_Strong, PM2_supporting, and PM3.

### 3.3 PGT and prenatal testing

**FIGURE 3:**
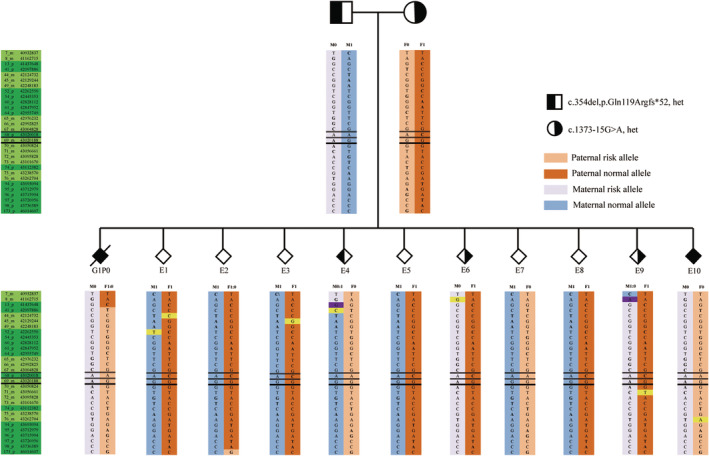
Results of haplotype linkage analysis.

Subsequently, the couple underwent a single PGT cycle, during which 10 embryos (E1–10) finally developed into blastocysts following ICSI. After TE biopsy, 5–8 cells were collected for whole‐genome amplification. Linkage analysis based on pedigree SNP haplotypes showed that E4 and E7 inherited only the paternal risk allele F0, whereas E6 and E9 inherited only the maternal risk allele M0, classifying them as carrier embryos. E10 inherited pathogenic variants from both alleles and was not recommended for transfer. E1–3, 5, 8, and 10 did not inherit parental risk alleles (Figure 3). Additionally, Sanger sequencing was performed on these 10 embryos (Figure S1), and the results were consistent with the haplotype outcomes.

Subsequent PGT‐A was performed in normal and carrier embryos. No CNVs larger than 4 Mb or aneuploidies were found in E3 or E5. E1 was identified as a mosaic embryo. E2 exhibited fragment deletions with mosaic signals. E4 and E9 were found to be aneuploids (Table 1).

**TABLE 1:** Summary of detection embryo results.

Embryo ID	Days	Embryo grade	PGT‐M haplotype	PGT‐M result	PGT‐A result
E1	D5	4BB	M1/F1	Normal	dup(mosaic)(1)(q43‐q44)(10.07 Mb)(65%)(238842212‐248908210)
E2	D5	4BB	M1/F1:0	Normal	del(mosaic)(2)(p25.3‐p11.2)(84.73 Mb)(68%)(10001‐84742692); del(2)(q14.2‐q37.3)(122.05 Mb)(121051338‐243102476); del(12)(p13.33‐q14.1)(58.11 Mb)(145740‐58256694); dup(mosaic)(12)(q14.1‐q24.33)(75.59 Mb)(42%)(58256695‐133841895)
E3	D5	4BB	M1/F1	Normal	Balanced euploid
E4	D5	4BB	M0:1/F0	Carrier of paternal risk allele	+1
E5	D5	4BB	M1/F1	Normal	Balanced euploid
E6	D5	4BC	M0/F1	Carrier of maternal risk allele	Balanced euploid
E7	D5	4BC	M1/F0	Carrier of paternal risk allele	Balanced euploid
E8	D6	4BC	M1/F1	Normal	Balanced euploid
E9	D6	4BC	M1:0/F1	Carrier of maternal risk allele	−15
E10	D6	4BC	M0/F0	High‐risk	—

E3, 5, and 8 were normal in both PGT‐M and PGT‐A and were recommended for transplantation. E3, with a morphological grade of 4BB on day 5, was selected for the first frozen embryo transfer cycle, considering the biopsy days and morphological grades of the embryos. This resulted in successful pregnancy.

Prenatal testing through amniocentesis was performed at 18 weeks of age using karyotyping, chromosomal microarray, and Sanger sequencing. No aneuploidy, CNV, or carrier status of the *CUL7* pathogenic variant was observed. A newborn with a birth weight of 3810 g and a length of 50 cm was delivered at 40 + 3 weeks.

## 4 DISCUSSION

Although 3M syndrome has been reported since 1975, no more than 200 cases have been reported to date. The involvement of *CUL7*, *OBSL1*, and *CCDC8* in 3M syndrome was first described in Huber et al. (2005), Hanson et al. (2009) and Hanson et al. (2011), respectively, but only 103, 40, and 3 variants related to 3M syndrome were identified in the HGMD database (professional 2022.4). 3M syndrome is characterized by severe pre‐ and postnatal growth restrictions, distinct facial features, and skeletal changes. Diagnosis based on facial characteristics and changes in the skeletal system can be made after birth. However, 3M syndrome remains underdiagnosed postnatally (Al‐Dosari et al., 2012). Prenatally, the presence of shortened long bones may suggest conditions such as osteogenic imperfecta or achondroplasia, which are not specific to 3M syndrome. Eight cases of 3M syndrome in fetuses have been diagnosed, and additional features have been reported in seven cases (Chincoli et al., 2014; Guo et al., 2020; Hu et al., 2017; Huang et al., 2023; Huber et al., 2009; Meo et al., 2000; Smogavec et al., 2022): two fetuses exhibited increased nuchal translucency (NT; Guo et al., 2020; Huang et al., 2023); two fetuses had a hypoplastic thorax; one had short nasal bones (Meo et al., 2000; Smogavec et al., 2022); one fetus had head frontal bossing and bilateral temporal bone depression (Hu et al., 2017); and one had characteristic facial features, prominent heels, and slender long bones (Huber et al., 2009). The increased NT should be distinguished from RASopathies, and most RASopathies do not exhibit severely short long bones during pregnancy. While increased NT and short nasal bones can be associated with trisomy 21 besides 3M syndrome. A hypoplastic thorax should be distinguished from short‐rib thoracic dysplasia. As fetal exome sequencing has been applied for prenatal diagnosis, studies have shown that skeletal anomalies yield approximately 40% of diagnostic results, among which achondroplasia (28.9%) and osteogenesis imperfecta (18.4%) are the most common finding (Huang et al., 2023). In our study, the fetus exhibited shortening of the long bones and nasal bones and lateral temporal bone depression. The mutation of FGFR3 c.1138 was not detected. Consequently, fetal WES was performed, which revealed two novel biallelic *CUL7* variants that could explain the prenatal ultrasound results and facilitate the prenatal diagnosis of 3M syndrome.

Exome sequencing and WES can only detect variants at the genome level, not at the transcriptional level. However, it is difficult to classify intronic variants of noncanonical splice sites as likely pathogenic or pathogenic without functional studies or previous reports. Abnormal splicing events can be detected at the transcriptional level using RT‐PCR, RNA‐seq, or minigenes. However, the transcriptome is specific to tissues/organs, and some genes cannot be detected in clinically accessible tissues such as blood, skin fibroblast cells, or simple tissue samples. Therefore, only variants with splicing damage potential in a particular group of genes can be validated using RT‐PCR or RNA‐seq (Ketkar et al., 2023). Minigenes can be used to investigate splice errors in vitro but may not reflect splicing events in vivo. Previously, only one noncanonical splice variant, c.3355+5G>A, was validated using minigenes. Among the variants identified in our study, c.1373‐15G>A was located in the intron, not in the canonical splice site. Based on the ACMG guidelines, this variant is difficult to classify as likely pathogenic or pathogenic. Although *CUL7* expression is relatively low at 4.11 TPM based on the GTEx database, we successfully performed RT‐PCR on RNA extracted from the mother's lymphocytes, rather than a minigene assay, and confirmed the aberrant splicing event in vivo. This enabled us to establish a method for detecting the aberrant splicing of *CUL7* using RNA from carriers. After aberrant splicing is confirmed, the variant is classified as likely pathogenic, and PGT can be performed.

Genetic counseling before the next pregnancy is highly recommended for couples with a history of adverse pregnancy outcomes. This will enable them to understand the risks associated with a family history and options available to them. 3M syndrome followed an autosomal recessive inheritance pattern, and the possibility of conceiving another affected fetus was 25%. In our study, the couple was confirmed to be carriers of likely pathogenic variants of *CUL7*, and during genetic counseling, they were informed of the 25% risk of having an affected fetus in future pregnancies. The counselor discussed the options for prenatal diagnosis, including chorionic villus sampling and amniocentesis, after natural conception, adoption, sperm donation, as well as electing not to reproduce, along with the alternative of PGT‐M. The couple would prefer to have a biologically related baby. Opting for PGT‐M, the couple successfully gave birth to a healthy child, avoiding the risk of conceiving an affected fetus.

In summary, we identified two novel variants of *CUL7*, expanding the mutation spectrum of *CUL7*, detecting aberrant splicing events of *CUL7* by RT‐PCR, and facilitated haplotype construction for PGT‐M. Collectively, these findings hold potential benefits for *CUL7* patients and their families.

## AUTHOR CONTRIBUTIONS

Xueqian Wang and Yi Huang designed this study. Xueqian Wang, Yaqiong He, and Xiaorong Wang performed the genetic and bioinformatic analyses. Xueqian Wang, Yunying Lin and Yi Huang prepared the figure and drafted the manuscript. Xiangtian Kong and Yejie Yao conducted the clinical evaluations and collected the clinical data. All the authors have read and approved the final manuscript.

## FUNDING INFORMATION

This study was supported by the National Natural Science Foundation of China (No. 82130046), National Key R&D Program of China (2019YFA0802604), the Science and Technology Project of Nantong City (No. JC2021082), the Municipal Health Commission of Nantong (No. MS2022079), Affiliated Maternity and Child Health Care Hospital of Nantong University (No. YYR202008), and the Jiangsu Health Innovation Team Program.

## CONFLICT OF INTEREST STATEMENT

The authors declare that they have no conflict of interest.

## Supporting information


Figure S1.

Table S1.

Caption

## Data Availability

Data supporting the findings of this study are available from the corresponding author upon request.
