# Supplementary figures and images for: Prenatal diagnosis and preimplantation genetics testing of 3M syndrome in a Chinese family with novel biallelic variants of CUL7
Source: Mol Genet Genomic Med. 2023 Oct 25;12(1):e2284. doi: 10.1002/mgg3.2284 (PMC10767403; doi:10.1002/mgg3.2284)

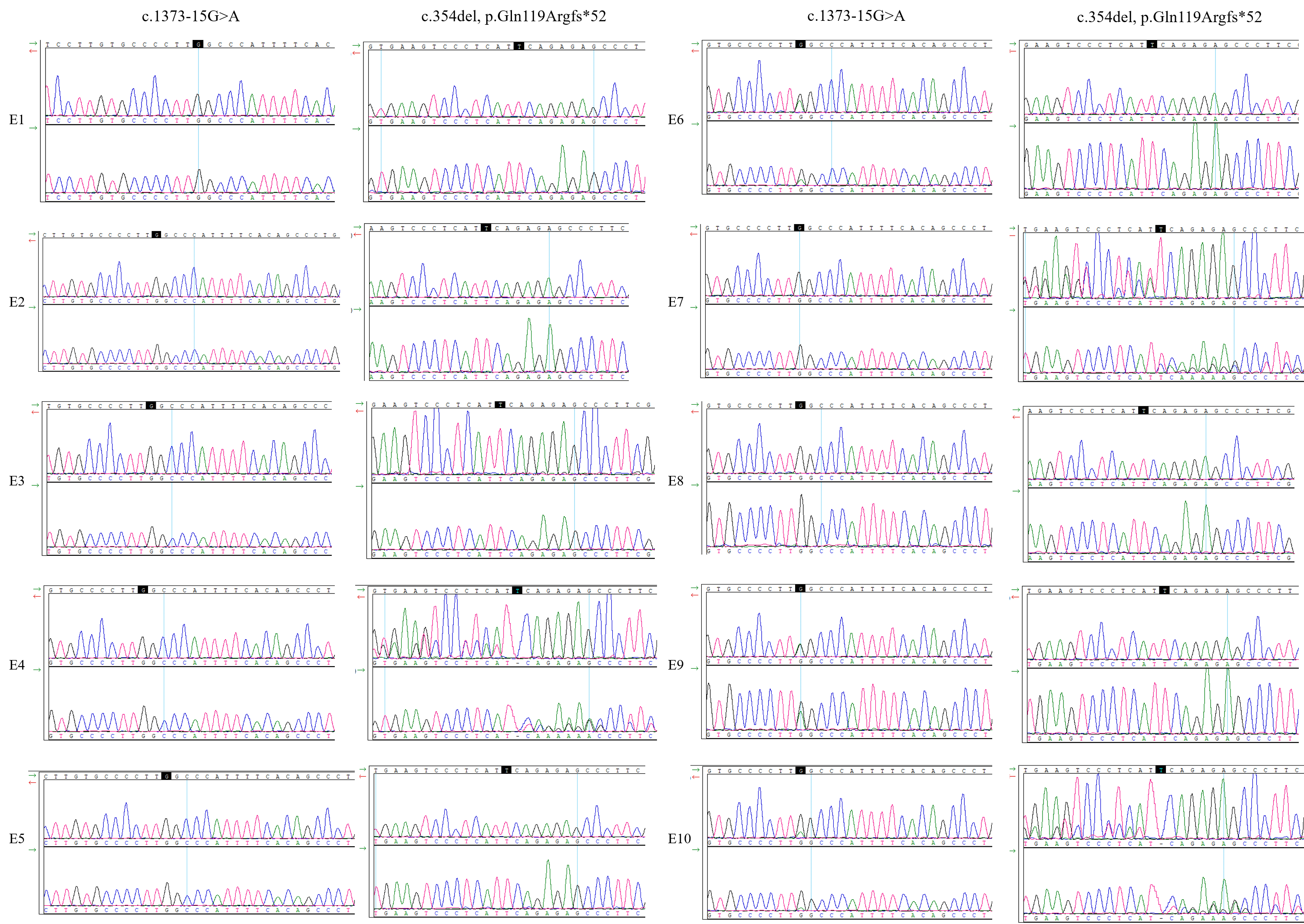

Supplement: Supplementary file 1 — Figure S1. [file MGG3-12-e2284-s003.tif]
