# Supplementary material for: Prenatal diagnosis and preimplantation genetics testing of 3M syndrome in a Chinese family with novel biallelic variants of CUL7
Source: Mol Genet Genomic Med. 2023 Oct 25;12(1):e2284. doi: 10.1002/mgg3.2284 (PMC10767403; doi:10.1002/mgg3.2284)
Supplement: Supplementary file 3 — Caption [file MGG3-12-e2284-s001.docx]

The caption for supplementary table 1 is “The primers used for haplotype construction” .

Supplementary Fig. 1 Sanger sequencing results of the *CUL7* variant in the embryos.
